# Walking Pattern Generation Through Step-by-Step Quadratic Programming for Biped Robots

**DOI:** 10.3390/biomimetics10100654

**Published:** 2025-10-01

**Authors:** Guoshuai Liu, Zhiguo Lu, Hang Zhang, Zeyang Liu

**Affiliations:** School of Mechanical Engineering and Automation, Northeastern University, Shenyang 110819, China; 2310102@stu.neu.edu.cn (G.L.); 2300486@stu.neu.edu.cn (H.Z.); 2000373@stu.neu.edu.cn (Z.L.)

**Keywords:** biped robot, walking pattern generation, height variation, quadratic optimization

## Abstract

The control of a biped robot is a challenging task due to the hard-to-stabilize dynamics. Generating a suitable walking reference trajectory is a key aspect of this problem. This article proposes a novel method of generating walking patterns for biped robots. The method integrates the double support phase and the single support phase into one step, and uses this step as the unit for trajectory generation through quadratic optimization with terminal constraints based on the Linear Inverted Pendulum Model, enabling us to shorten the optimization horizon while still generating natural walking trajectories. Moreover, by restricting the position and acceleration of the center of mass (COM) in the vertical direction, an excessive constraint is formed on the Zero Moment Point (ZMP) to offset the nonlinear effects of the COM’s vertical motion on the ZMP. This allows the COM of the robot to change in the vertical direction while maintaining the linearity of the optimization problem. Finally, the performance of the proposed method is validated by simulations and experiments of walking on flat ground and stairs using a position-controlled biped robot, Neubot.

## 1. Introduction

The control of a biped robot is a challenging task due to its difficult-to-stabilize dynamics. Generating a suitability walking reference trajectory is a key aspect of this problem [1]. The walking trajectory mainly includes the motion trajectories of both feet and the center of mass (COM). To ensure the robot walks stably, the Zero Moment Point (ZMP) determined by the COM state needs to be located within the support polygon, which is the convex hull of the contact points between the feet and the ground. Due to the alternating contact of the two feet with the ground, the support polygon experiences step-like changes when the swinging leg lands or the supporting leg lifts up. However, the change in the COM is continuous, so when planning the COM trajectory, it is necessary to predict these step-like changes in advance to meet the ZMP stability criterion. Kajita et al. [2] generated the trajectory of the COM through preview control of the ZMP, which anticipates future foot placements to optimize stable and efficient motion. This approach was further developed into Model Predictive Control (MPC) to achieve even more stable walking motions under strong perturbations [3,4,5]. However, to ensure the reliability of this method, the preview horizon is set to at least two step periods, which increases computational costs.

Khadiv et al. [6] argue that it is not necessary to optimize walking over several steps to ensure gait viability and show that it is sufficient to merely select the next step timing and location. And enforcing a reasonable terminal constraint (i.e., a constraint on the COM’s state at the end of the optimization horizon) can ensure capturability, which can be used to shorten the predictive horizon. The Munich University Johnny robot [7] enforced a terminal constraint on the position of the COM at the end of the next two steps. The Honda Asimo robot [8] imposed cyclicity of motion by constraining the divergent component of the dynamics at the end of the next step, which was a true capturability constraint. The walking and running motion generation scheme implemented in the Toyota Partner robot [9] imposed cyclicity of motion through both the position and velocity of the COM. In this article, we impose a terminal constraint on the position, velocity, and acceleration of the COM at the end of the current step, meaning that the walking trajectory is generated within a single step period. When the terminal constraints are properly set, a refined walking trajectory can be optimized.

When using quadratic optimization to generate walking trajectories, there are primarily two approaches to handle the ZMP. One approach is to include the ZMP in the objective function to minimize the deviation of the ZMP from the center of the foot sole [2,10,11], or to align the ZMP trajectory with a reference trajectory from heel to toe [12,13,14]. However, the specified reference points and trajectories often do not match the natural walking pattern, and there is no strict guarantee that the ZMP will remain within the support surface. Another approach is to include the ZMP in the constraint conditions, thus strictly satisfying the ZMP criteria. The support polygon expands during the transition from the single support phase to the double support phase, resulting in corresponding changes to the ZMP boundary conditions. However, despite this, refs. [15,16] only considered the single support phase during the whole walking process. This is because the ZMP movement constraints applied in the single support phase are more restrictive and the sampling time can be large enough to omit the double support phase in the predictive framework. But ignoring the ZMP in the double support phase can cause the acceleration of the COM to be discontinuous and lack naturalness. In this article, we propose a novel step period that includes both single and double support phases to generate a continuous moving ZMP trajectory.

To efficiently generate walking patterns, simplified dynamic models have been developed to approximate complex robot systems, with the Linear Inverted Pendulum Model (LIPM) being the most widely used [17]. However, to ensure the system remains linear, the LIPM confines the COM to a horizontal plane, as the relationship between the vertical COM state and the ZMP is nonlinear. To enable variations in the COM height, some work [18,19,20] incorporated nonlinear components and used the Sequential Quadratic Programming (SQP) method to solve the Nonlinear Model Predictive Control (NMPC) problem, generating the walking trajectory. Reference [21] treated the nonlinear components as time-varying quantities and formulated the preview control problem as a Linear Quadratic Regulator (LQR) problem for time-varying linear systems to generate walking trajectories. Whereas the above methods achieve variations in the COM height by increasing computational costs, ref. [22] bound the nonlinear component within suitable extreme values. By ensuring that the ZMP constraints were satisfied at these extreme values, it guaranteed that all intermediate values also satisfied the constraints. This method converted the nonlinear problem into a linear one by overly constraining the ZMP. But in [22], the selection criteria for extreme values were not specifically explained. Additionally, the nonlinear component was constrained as a whole without specific consideration of its individual variables. In this article, we impose boundary constraints on the vertical state of the COM, including its position and acceleration, according to the robot’s motion conditions. This ensures the optimization problem remains linear while accommodating variations in COM height.

In this article, we focus on the generation of walking patterns. The main contributions of this work are as follows.

(1) We propose a novel step period that includes both single and double support phases, and generate walking trajectories in units of a step by imposing terminal constraints. This approach allows us to shorten the optimization horizon while still generating natural walking trajectories.
(2) We impose boundary constraints on the vertical state of the COM, ensuring the linearity of the optimization problem while allowing for variable COM height.

**Figure 1:**
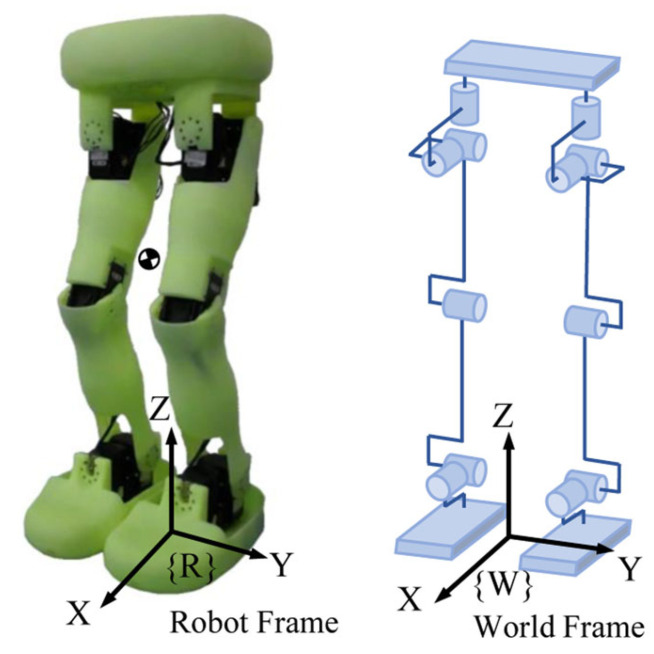
Neubot, a position-controlled biped robot. It has 12 degrees of freedom, stands at a height of 0.57 m, and weighs 3.1 kg.

The rest of this article is organized as follows. Section 2 introduces the motion model and the novel step. In Section 3, the generation of walking patterns for flat ground is introduced. In Section 4, the generation of walking patterns with variable COM height is introduced. In Section 5, we introduce the whole control frame. In Section 6, the method is demonstrated by numerical comparisons, simulations, and experiments conducted on the robot Neubot, as shown in Figure 1. Finally, Section 7 sets out the conclusions for this article.

## 2. Preliminaries

### 2.1. Motion Model and Constraints

In biped robot systems, the combination of nonlinear, underactuated, and high-dimensional dynamics results in a particularly challenging control problem. Therefore, reduced-order models, such as the LIPM for walking [23] and the Spring-Loaded Inverted Pendulum (SLIP) model for running [24], have been widely applied for controlling the locomotion of legged robots. In this article, we contract the robot to a point mass (i.e., COM) and disregard the inertial effects caused by rotations of its various parts. When only considering the horizontal motion of the COM, the dynamic system along the *X*-axis can be expressed as

(1) $$\frac{d}{dt}\left[\begin{array}{c} x\\ \dot{x}\\ \ddot{x} \end{array}\right]=\left[\begin{array}{ccc} 0 & 1 & 0\\ 0 & 0 & 1\\ 0 & 0 & 0 \end{array}\right]\left[\begin{array}{c} x\\ \dot{x}\\ \ddot{x} \end{array}\right]+\left[\begin{array}{c} 0\\ 0\\ 1 \end{array}\right]u_{x}\;\;\frac{d}{dt}\ddot{x}=u_{x}$$

where x is the position of the COM on the *X*-axis, and $u_{x}$ denotes the input of the system.

To ensure the robot walks stably, it is sufficient for the Center of Pressure (COP), also known as the ZMP, to lie within the support polygon. Therefore, the constraint condition is

(2) $$p_{x}=\left[\begin{array}{ccc} 1 & 0 & -\frac{z-p_{z}}{\ddot{z}+g} \end{array}\right]\left[\begin{array}{c} x\\ \dot{x}\\ \ddot{x} \end{array}\right]\;\;p_{x}\in S$$

where $p_{x}$ is the ZMP position on the *X*-axis, which can be considered as the output of the dynamic system; z and $p_{z}$ represent the vertical heights of the COM and the support foot, respectively; g is the gravity acceleration; and c is the support polygon.

In the above, only the horizontal position of the COM along the *X*-axis is expressed, but the same applies to the horizontal position along the *Y*-axis.

### 2.2. A Step Period with Double Support

**Figure 2:**
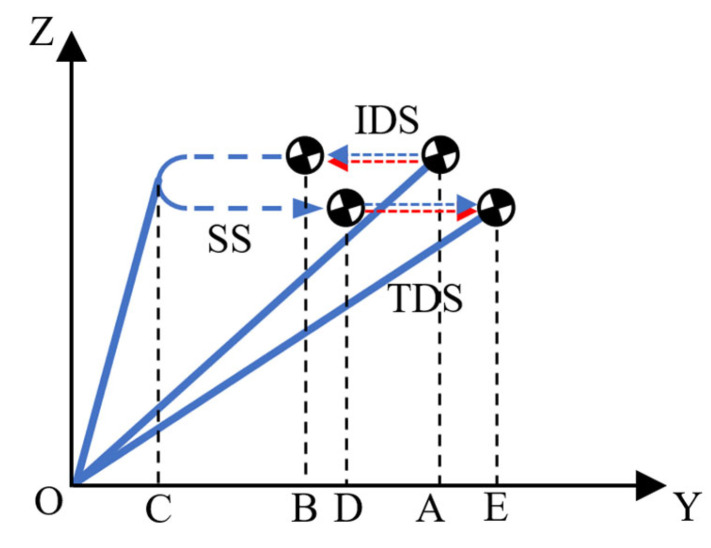
A support period of one leg. The blue line represents the COM trajectory during the left-leg support period, the red line during the right-leg support period, and their overlap indicates the trajectory during the double-support period.

**Figure 3:**
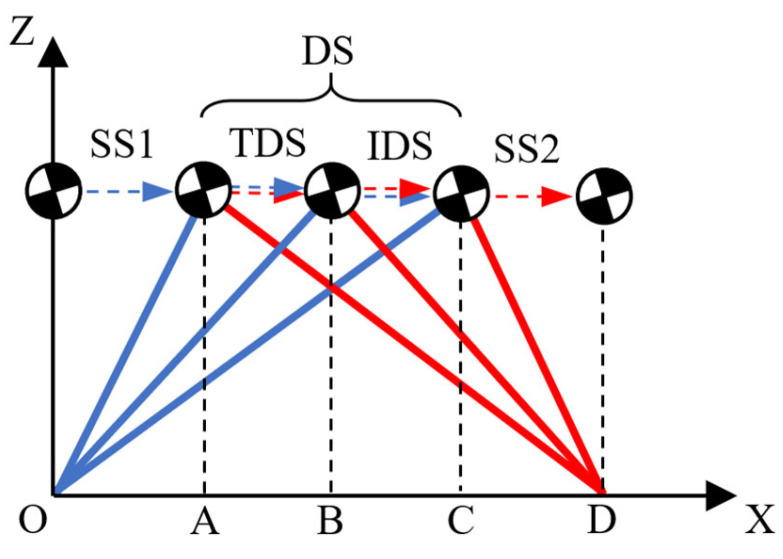
A step period for the COM in the sagittal plane. The blue line represents the COM trajectory during the left-leg support period, the red line during the right-leg support period, and their overlap indicates the trajectory during the double-support period.

In this article, the generation of walking patterns is conducted in units of steps. Here, we will first introduce the concept of a novel step. In [25], the gait cycle of a human limb can be divided into different phases, as shown in Table 1. According to this, the support period of one leg can be divided into three stages, namely, initial double support (IDS), single support (SS), and terminal double support (TDS), as shown in Figure 2. A step period, as defined in this article, comprises the latter half of the prior support period (i.e., SS1 and TDS) and the first half of the following support period (i.e., IDS and SS2), as shown in Figure 3. The support period is primarily for motion planning of the COM in the lateral plane, while the step period is mainly for motion planning of the COM in the sagittal plane. It should be emphasized that the origin of the robot coordinate frame is defined beneath the center of the current supporting foot, as shown in Figure 1, and the trajectory is generated in robot frame {R} and analyzed in world frame {W}.

## 3. Walking Pattern Generation for a Step

In this section, we generate motion patterns for a robot walking on flat ground, with the assumption that the COM height remains constant. Therefore

(3) $$p_{z}=0\;\;z=z_{c}\;\;(\mathrm{constant})\;\;\ddot{z}=0$$

And then Equation (2) can be written as

(4) $$p_{x}=\left[\begin{array}{ccc} 1 & 0 & -\frac{z_{c}}{g} \end{array}\right]\left[\begin{array}{c} x\\ \dot{x}\\ \ddot{x} \end{array}\right]$$

Building upon this premise, quadratic programming is employed to generate the COM motion trajectory in the sagittal plane within a step period. Similarly, in the lateral plane, the COM motion trajectory is determined using quadratic programming over a support period.

### 3.1. Quadratic Programming Within a Step

First, a walking pattern generation for sagittal plane is described. The dynamic Equations (1) and (2) at discrete time $t_{k}$ can be reformulated in a discrete form for the controller interval △t, as

(5) $${\hat{x}}_{k+1}=A{\hat{x}}_{k}+Bu_{xk}\;\;p_{xk}=\left[1\;\;\;\;0\;\;\;\;-z_{c}/g\right]{\hat{x}}_{k}$$

where

$${\hat{x}}_{k}=\left[\begin{array}{c} x(t_{k})\\ \dot{x}(t_{k})\\ \ddot{x}(t_{k}) \end{array}\right]\cdot \;u_{xk}=u_{x}(t_{k})$$

$$A=\left[\begin{array}{ccc} 1 & △t & △t^{2}/2\\ 0 & 1 & △t\\ 0 & 0 & 1 \end{array}\right]\cdot \;B=\left[\begin{array}{c} △t^{3}/6\\ △t^{2}/2\\ △t \end{array}\right]$$

We compute the input $u_{xk}$ of the dynamic system (5) that minimizes the following function over a step period:

(6) $$\begin{array}{l} \underset{u_{x0},u_{x1},\ldots u_{xn-1}}{\min}\sum_{k=0}^{n-1}\frac{\alpha }{2}{\left\|u_{xk}\right\|}^{2}+\frac{\beta }{2}{\left\|{\ddot{x}}_{k}\right\|}^{2}+\frac{\gamma }{2}{\left\|{\dot{x}}_{k}-{\dot{x}}_{k}^{ref}\right\|}^{2}\\ s.t.\\ \;\;\;\;\;\;\;{\hat{x}}_{k+1}=A{\hat{x}}_{k}+Bu_{xk}\\ \;\;\;\;\;\;\;p_{xk}=\left[\begin{array}{ccc} 1 & 0 & -z_{c}/g \end{array}\right]{\hat{x}}_{k}\\ \;\;\;\;\;\;\;-p_{x}^{lim}\leq p_{xk}\leq p_{x}^{lim}\left(t_{k}\in \mathrm{SS}1\right)\\ \;\;\;\;\;\;\;-p_{x}^{lim}\leq p_{xk}\leq p_{x}^{foot\_next}+p_{x}^{lim}\left(t_{k}\in \mathrm{DS}\right)\\ \;\;\;\;\;\;\;p_{x}^{foot\_next}-p_{x}^{lim}\leq p_{xk}\leq p_{x}^{foot\_next}+p_{x}^{lim}\left(t_{k}\in \mathrm{SS}2\right)\\ \;\;\;\;\;\;\;{\ddot{x}}_{n}=0\\ \;\;\;\;\;\;\;{\dot{x}}_{n}={\dot{x}}_{n}^{ref}\\ \;\;\;\;\;\;\;x_{n}=p_{x}^{foot\_next} \end{array}$$

where n is the horizon length in a step period, $p_{x}^{foot\_next}$ is the next foothold point in the *X*-axis, and $p_{x}^{lim}$ represents the support range of the feet in the *X*-axis.

In Equation (6), the first term avoids jitter in the forces acting on the COM, the second term minimizes the forces acting on the COM, and the third term makes the COM velocity approach the reference velocity. The first constraint represents the system’s state equation. The second to fifth constraints ensure that the ZMP remains within S throughout the entire step period. The sixth and seventh constraints impose constraints on the terminal state of the step period, ensuring that the COM arrives directly above the next foothold point $p_{x}^{foot\_next}$ with the desired velocity ${\dot{x}}_{n}^{ref}$ at the end of a step period. These constraints also ensure that the ZMP is located at $p_{x}^{foot\_next}$ by the end of the step period. When the robot needs to stop, ${\dot{x}}_{n}$ is set to 0. After determining $u_{xk}$ with Equation (6), the trajectory of the COM along the *X*-axis can be obtained using Equation (5).

Similarly, the trajectory of the COM along the *Y*-axis can be obtained by

(7) minuy0,uy1,…uyn−1∑k=0n−1α2uyk2+β2y¨k2+γ2y˙k2s.t.        y ^k+1=Ay^k+Buyk       pyk=10−zc/gy^k       −pylim≤(−1)spyk≤(−1)spyfoot_last+pylimtk∈IDS       −pylim≤pyk≤pylimtk∈SS       −pylim≤(−1)spyk≤(−1)spyfoot_next+pylimtk∈TDS       y¨n=0        y ˙n=0       yn=pyfoot_next/2

where s indicates whether the support is from the left or right foot, with s=0 for left foot support and s=1 for right foot support. $p_{y}^{foot\_last}$ and $p_{y}^{foot\_next}$ represent the positions of the last and the next support foot along the *Y*-axis, respectively. It should be noted that the seventh constraint is only used as a constraint when the robot needs to stop. And the eighth constraint ensures that the COM is positioned at the center between the two supporting feet along the *Y*-axis at the end of the support period.

During the double support phase, the constraint polygon for the ZMP defined by the third constraint and the fifth constraint is larger than the support polygon. However, the subsequent results show that the ZMP still remains within the support polygon.

### 3.2. Foot Placement

In generating the COM trajectory, the position of the next support foot $\left(p_{x}^{foot\_next},p_{y}^{foot\_next}\right)$ is involved. It is determined by

(8) $$p_{x}^{foot\_next}=v_{x}^{d}T$$

$$p_{y}^{foot\_next}=2v_{y}^{d}T+{\left(-1\right)}^{s}d$$

where T represents the duration of a step period and a support period, which remains constant throughout the walk. $v_{x}^{d}$ and $v_{y}^{d}$ are the desired walking speeds of the robot in the *X*-axis and *Y*-axis directions, respectively. And d is the length of the robot pelvis (the default step width).

## 4. Walking Pattern Generation with Height Variation

In the previous section, we generated a trajectory for robots walking on flat ground with the COM constrained to a fixed height. In this section, we will remove this constraint and generate a walking pattern that allows for variable COM heights.

### 4.1. Constraints on the ZMP with Variable COM Height

**Figure 4:**
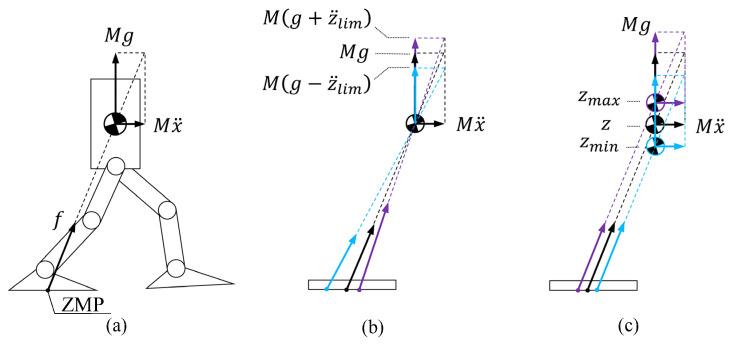
Relationship between the COM’s motion and the ZMP: (**a**) the COM is maintained at a fixed height; (**b**) the COM has vertical acceleration; (**c**) the COM has height variation in the vertical direction. Arrows and dashed lines indicate the direction of the applied force.

When the robot’s COM moves at a constant height, as shown in Figure 4a, the ground reaction force f passing through the ZMP balances with the gravity compensation force Mg and the acceleration force $M\ddot{x}$. When the COM has an acceleration in the vertical direction, the change in ZMP is as shown in Figure 4b. When the COM moves in the vertical direction, the change in ZMP is as shown in Figure 4c. Therefore, by limiting the range of the COM’s position and acceleration, we can determine the range of ZMP variation and maintain the stability of the robot by ensuring that the boundary values of ZMP remain within the support polygon. In this manner, we can vary the COM height while preserving the linearity of the optimization problem, though it may excessively restrict the ZMP.

Next, determine the boundary values of the ZMP. Equation (5) can be written as

(9) $$p_{x}\left(z,\ddot{z}\right)=x-\frac{z-p_{z}}{\ddot{z}+g}\ddot{x}$$

This function only considers the influence of z and $\ddot{z}$ on the ZMP position. And the range of the variables z and $\ddot{z}$ is defined as

(10) $$z_{min}\leq z\leq z_{max}\;-{\ddot{z}}_{lim}\leq \ddot{z}\leq {\ddot{z}}_{lim}$$

So, when $\ddot{x}\geq 0$,

(11) $$p_{x}\left(z_{max},-{\ddot{z}}_{lim}\right)\leq p_{x}\left(z,\ddot{z}\right)\leq p_{x}\left(z_{min},{\ddot{z}}_{lim}\right)$$

And, when $\ddot{x}\leq 0$,

(12) $$p_{x}\left(z_{min},{\ddot{z}}_{lim}\right)\leq p_{x}\left(z,\ddot{z}\right)\leq p_{x}\left(z_{max},-{\ddot{z}}_{lim}\right)$$

During the trajectory optimization process, simultaneously consider both (11) and (12), without determining the sign of $\ddot{x}$.

### 4.2. Quadratic Programming with Height Variation

After obtaining the boundary value of ZMP determined by the variation range of the COM’s position and acceleration in the vertical direction, the three-dimensional trajectory of the COM can be generated. First, determine the COM’s trajectory along the *X*-axis and *Z*-axis by

(13) $$\begin{array}{l} \underset{\begin{array}{l} u_{x0},u_{x1},\ldots u_{xn-1}\\ u_{z0},u_{z1},\ldots u_{zn-1} \end{array}}{\min}\sum_{k=0}^{n-1}\frac{\alpha }{2}({\left\|u_{xk}\right\|}^{2}+{\left\|u_{zk}\right\|}^{2})+\frac{\beta }{2}({\left\|{\ddot{x}}_{k}\right\|}^{2}+{\left\|{\ddot{z}}_{k}\right\|}^{2})+\frac{\gamma }{2}({\left\|{\dot{x}}_{k}\right\|}^{2}+{\left\|{\dot{z}}_{k}\right\|}^{2})\;\\ s.t.\\ \;\;\;\;\;\;\;{\hat{x}}_{k+1}=A{\hat{x}}_{k}+Bu_{xk}\\ \;\;\;\;\;\;\;{\hat{z}}_{k+1}=A{\hat{z}}_{k}+Bu_{zk}\\ \;\;\;\;\;\;\;p_{xk}\left(z,\ddot{z}\right)=x_{k}-\frac{z-p_{zk}}{\ddot{z}+g}{\ddot{x}}_{k}\\ \;\;\;\;\;\;\;-p_{x}^{lim}\leq p_{xk}\left(z_{max},-{\ddot{z}}_{lim}\right)\leq p_{x}^{lim}\left(t_{k}\in \mathrm{SS}1\right)\\ \;\;\;\;\;\;\;-p_{x}^{lim}\leq p_{xk}\left(z_{min},{\ddot{z}}_{lim}\right)\leq p_{x}^{lim}\left(t_{k}\in \mathrm{SS}1\right)\\ \;\;\;\;\;\;\;p_{x}^{foot}-p_{x}^{lim}\leq p_{xk}\left(z_{max},-{\ddot{z}}_{lim}\right)\leq p_{x}^{foot\_next}+p_{x}^{lim}\left(t_{k}\in \mathrm{SS}2\right)\\ \;\;\;\;\;\;\;p_{x}^{foot}-p_{x}^{lim}\leq p_{xk}\left(z_{min},{\ddot{z}}_{lim}\right)\leq p_{x}^{foot\_next}+p_{x}^{lim}\left(t_{k}\in \mathrm{SS}2\right)\\ \;\;\;\;\;\;\;{\ddot{x}}_{n}=0\\ \;\;\;\;\;\;\;{\dot{x}}_{n}={\dot{x}}_{n}^{ref}\\ \;\;\;\;\;\;\;x_{n}=p_{x}^{foot\_next} \end{array}$$

where, unlike (6), it includes the trajectory planning of COM in the *Z*-axis direction, and restricts the ZMP position according to (11) and (12) in the SS1 and SS2 phases. However, during the DS phase, the two supporting feet are not at the same height, which increases the difficulty of calculating the ZMP. To simplify the approach, no constraints are applied to the ZMP during this phase, similar to [5] And it should also be emphasized that when the robot needs to stop, ${\dot{x}}_{n}$ is set to 0.

Secondly, determine the trajectory of COM in the *Y*-axis direction by

(14) minuy0,uy1,…uyn−1∑k=0n−1α2uyk2+β2y¨k2+γ2y˙k2s.t.        y ^k+1=Ay^k+Buyk       pyk=y−zk−pzz¨k+gy¨       −pylim≤pyk≤pylimtk∈SS       y¨n=0       y ˙n=0       yn=pyfoot_next/2

where the second constraint takes into account the change in $z_{k}$ and ${\ddot{z}}_{k}$, which are derived from (13). Similarly, the fifth constraint is only used as a constraint when the robot needs to stop.

## 5. Overview of the Control Framework

**Figure 5:**
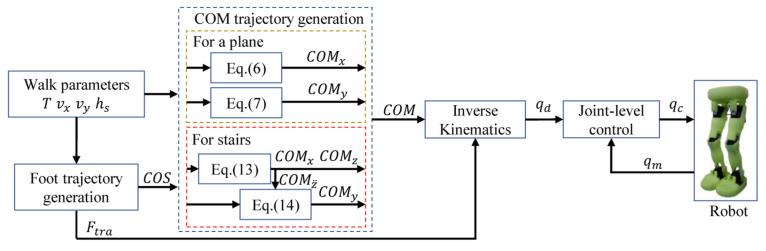
Overall control framework of biped robot for walking pattern generation.

Figure 5 shows the overall framework of the robot, primarily focusing on the generation of the COM trajectory for a plane or stairs. First, based on the given walk parameters such as walking period T, walking speeds $v_{x}$ and $v_{y}$, and step height $h_{s}$, generate the foot trajectory $F_{tra}$ and the foothold COP. Secondly, based on walk parameters and COP, generate COM trajectory COM. For a plane, (6) optimizes the trajectory COMx for the *X*-axis, (7) optimizes the trajectory COMy for the *Y*-axis, and COMz is constant. For stairs, (13) optimizes the trajectories COMx and COMz for the *X*-axis and *Z*-axis, respectively, and (14) optimizes the trajectory COMy for the *Y*-axis. Third, the desired joint angles $q_{d}$ are obtained through the inverse kinematics to realize the desired COM and $F_{tra}$. Lastly, perform position control on the robot at the joint level by control angles $q_{c}$ and measure angles $q_{m}$.

## 6. Method Demonstrations

### 6.1. Numerical Comparison

To intuitively show the advantage of our proposed walking pattern generation method, numerical comparisons were implemented. We used three different methods to generate the COM trajectory along the *X*-axis within one step based on the LIPM. The specified walking parameters are detailed in Table 2.

These three methods utilized the same objective function as shown in (6) but differed in the constraints applied, as follows:

Method 1: Optimize the COM trajectory over a two-step horizon without terminal constraints or the double support phase.

Method 2: Optimize the COM trajectory over a one-step horizon with terminal constraints, as shown in (6), but without the double support phase.

Method 3: Optimize the COM trajectory over a one-step horizon with terminal constraints and the double support phase, as shown in (6). And the proportions for the three stages SS1, DS, and SS2 are 10%, 80%, and 10%, respectively, based on the human gait analysis reported in [25], which reflects the typical durations of single support and double support phases during walking.

**Figure 6:**
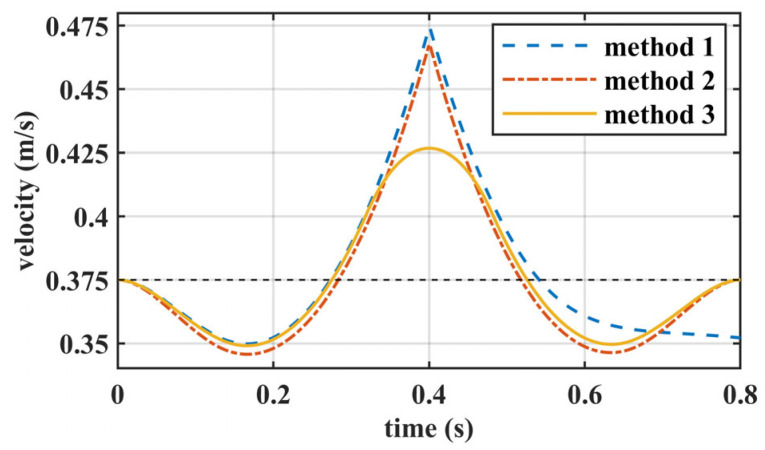
The velocity trajectory for a step using different methods.

**Figure 7:**
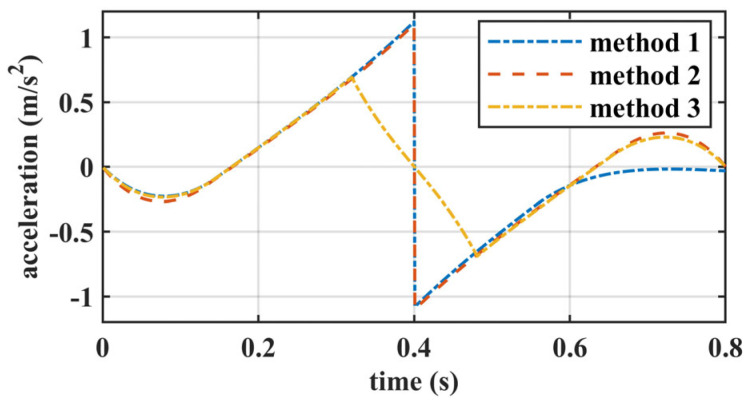
The acceleration trajectory for a step using different methods.

By setting the initial, desired, and terminal speeds to 0.375 m/s, the COM trajectory was optimized using these three methods. The resulting COM velocities are shown in Figure 6, and the resulting COM accelerations are shown in Figure 7. Figure 6 illustrates that due to the terminal constraints on the COM state, both Method 2 and Method 3 achieve the desired speed of 0.375 m/s at the end of one step. In contrast, Method 1 deviates from the desired speed at the end of one step. Furthermore, due to the presence of the double support phase, Method 3 deviates less from the desired speed within one step compared to Method 1 and Method 2. As shown in Figure 7, there are abrupt changes in acceleration in Method 1 and Method 2, due to the instantaneous exchange of the support leg with the swing leg. In Method 3, however, there is a smooth transition in acceleration during the double support phase, moving smoothly from the acceleration state to the deceleration state.

**Figure 8:**
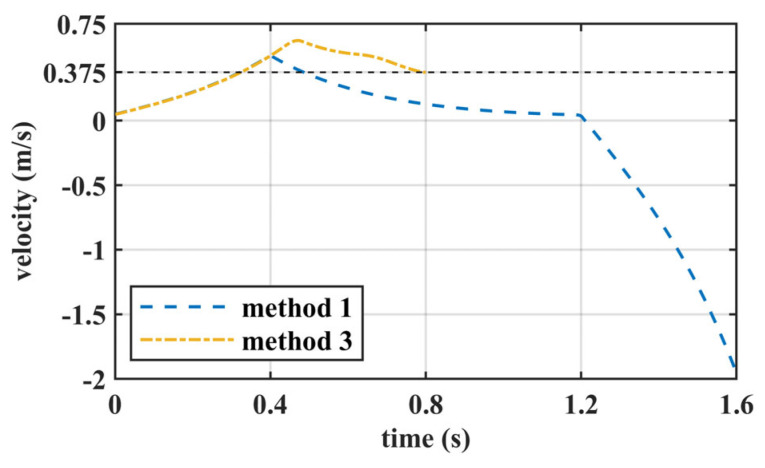
The optimized velocity trajectory with an initial velocity of 0.05 m/s.

When the initial velocity decreases to 0.05 m/s and the desired and terminal velocities are maintained at 0.375 m/s, the velocity of the generated COM trajectory is as shown in Figure 8. At the end of a step, Method 3 is able to reach the desired speed, while Method 1 deviates significantly from the desired speed. In addition, it can be found that Method 1 will be in a divergent state in the two steps. For Method 2, optimization did not converge, and no COM trajectory was generated. This is because the initial speed is small, and the COM cannot achieve a sufficient speed during the acceleration phase to meet the terminal constraints.

In conclusion, comparing Method 1 and Method 2 reveals that reasonable terminal constraints can achieve an ideal COM trajectory while shortening the optimized horizon. Furthermore, adding a double support phase to Method 2 transforms it into Method 3. Compared to Method 2, Method 3 demonstrates the capability to achieve a smooth transition of acceleration which is crucial for the stability of the robot.

### 6.2. Walk Simulation on Flat Ground

In the Webots simulation environment, we conducted simulations of walking on flat ground.

**Figure 9:**
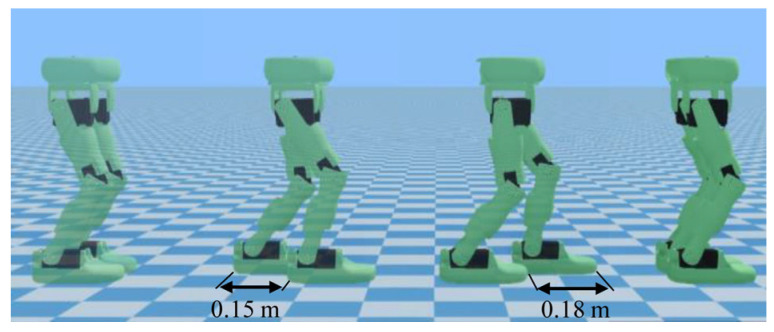
Simulation experiment for walking in a straight line.

The first simulation is the robot walking in a straight line along the *X*-axis, as shown in Figure 9. The step length is 0.15 m in the first half and increases to 0.18 m in the second half, with a speed transition process in between. The specified walking parameters are detailed in Table 3.

**Figure 10:**
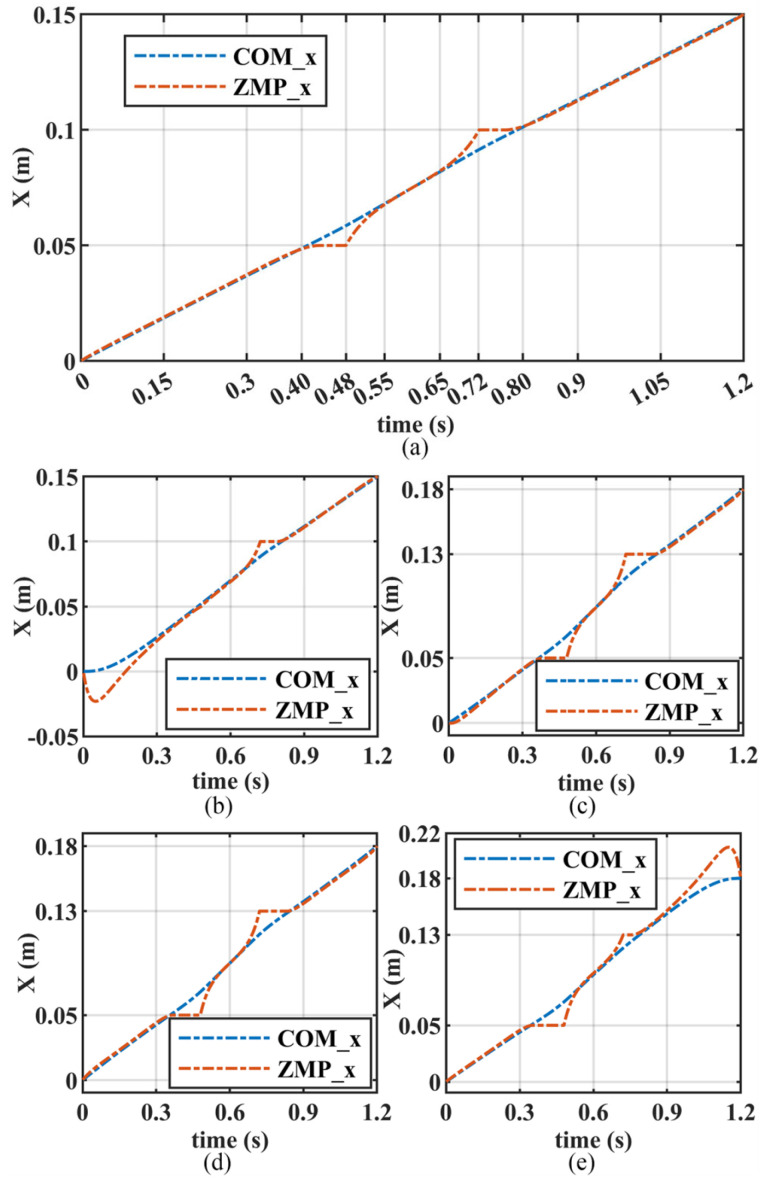
The COM and ZMP trajectories for different steps: (**a**) the uniform speed step with an initial and terminal constraint velocity of 0.125 m/s, and a step length of 0.15 m; (**b**) the startup step with an initial velocity of 0 m/s, terminal constraint velocity of 0.125 m/s, and a step length of 0.15 m; (**c**) the variable speed step with an initial velocity of 0.125 m/s, terminal constraint velocity of 1.5 m/s, and a step length of 0.18 m; (**d**) the uniform speed step with an initial and terminal velocity of 0.15 m/s, and a step length of 0.18 m; (**e**) the stopping step with an initial velocity of 0.15 m/s, terminal constraint velocity of 0 m/s, and a step length of 0.18 m.

Figure 10 illustrates the COM and ZMP trajectories generated through step optimization for different initial and terminal velocities. Figure 10a shows the step of the robot walking at an approximate uniform speed with a step length of 0.15 m. At 0 s, the COM and ZMP are both located in the middle of the support foot. Subsequently, they move forward at nearly the same speed. At 0.40 s, the ZMP reaches 0.05 m, which is the front edge of the support surface. Afterwards, the COM continues to move forward while the ZMP stays at 0.05 m. In this case, the COM can be regarded as performing linear inverted pendulum motion at the support point of 0.05 m. At 0.48 s, the robot enters the double support phase, and the ZMP begins to approach the COM. From 0.55 s to 0.65 s, the trajectories of the COM and ZMP almost coincide. Subsequently, the ZMP moves ahead of the COM, and the COM enters a deceleration state. At 0.72 s, the robot enters the single support phase, supported by the next foot, and the ZMP reaches 0.1 m, which is the rear edge of the next support surface. Then, the ZMP remains at this position until it coincides with the COM. Finally, the COM and ZMP move along almost identical trajectories to the middle of the next support foot.

Figure 10b shows the step of the robot startup phase. The ZMP lags behind COM in the early stage, putting COM in an accelerated state. This allows the robot to gain a certain speed. Figure 10c shows the step of the robot with variable speed and step length. The initial velocity is 0.125 m/s, the terminal velocity is 0.15 m/s, and the step length is 0.18 m. The shape of the COM and ZMP trajectories in this step is similar to those in Figure 10a. The difference is that in the early stage of this step, the ZMP is behind the COM. This is due to the COM needing to increase from 0.125 m/s to 0.15 m/s. Figure 10d shows the step of the robot walking at an approximate uniform speed with a step length of 0.18 m. Different from Figure 10a, in this step, the ZMP lingers longer at the edge of the support surface at 0.05 m and 0.13 m. This is due to the increase in step length, or more precisely, the increase in speed. Figure 10e shows the step of the robot stopping phase. In contrast to the process in Figure 10b in the later stages of this step, the ZMP is ahead of the COM, causing the COM to decelerate to zero. Finally, the COM and ZMP are both located in the middle of the support surface.

Furthermore, from Figure 10, it can be observed that the ZMP trajectory generated by the step optimization proposed in this article conforms the pattern of human walking. In the SS1 phase, the ZMP moves from the middle of the support surface to the front edge. During the DS phase, the ZMP shifts from the front edge of the support surface to the rear edge of the next foot support. In the SS2 phase, the ZMP moves from the rear edge of the next foot support to its middle. Here, the front edge of the support surface corresponds to the toes, and the rear edge corresponds to the heel. Additionally, the change in relative positions between COM and ZMP allows for flexible adjustment of COM velocity.

**Figure 11:**
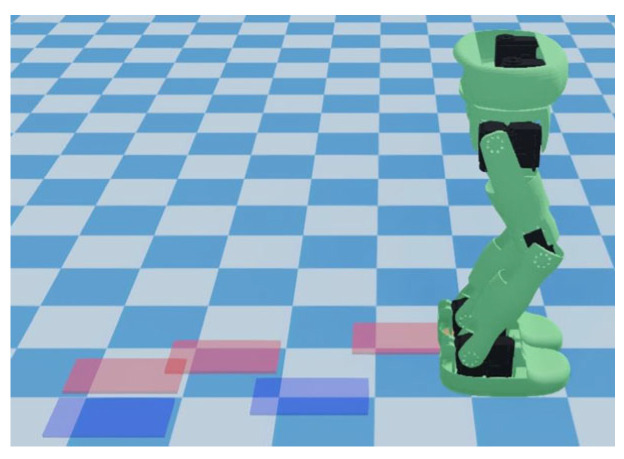
Simulation experiment of diagonal walking.

**Figure 12:**
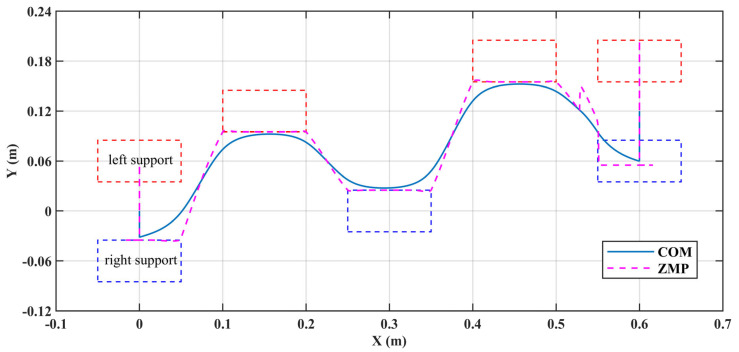
The foot placement distribution, along with the COM and ZMP trajectories, for diagonal walking. Note that the support polygon is smaller than the actual foot surface for a safety margin.

In the second demonstration, the robot performed diagonal walking, as shown in Figure 11. The specified walking parameters are detailed in Table 4. The distribution of foot placements and the trajectory of the COM and ZMP are illustrated in Figure 12. During the single support phase, the ZMP trajectory approaches the inner edge of the support surface, thereby reducing the lateral sway amplitude of the COM. In the double support phase, ZMP transitions from one support surface to another in an approximately straight trajectory, which is an ideal transition trajectory. Additionally, Figure 12 also illustrates that the COM trajectory is relatively smooth and closely follows the ZMP trajectory, which contributes to the stability of the robot.

It should be emphasized that during the startup phase, the COM moves in the lateral plane for half a step period before starting to move in the sagittal plane, which causes the ZMP to move along the *Y*-axis first. The opposite is true during the stopping phase. Additionally, in the final support period, the ZMP is discontinuous along the *Y*-axis and suddenly shifts to the left, as shown in Figure 12. This adjustment ensures that the COM moves further to the right, allowing it to come to a stop between the two feet at the end of this support period.

**Figure 13:**
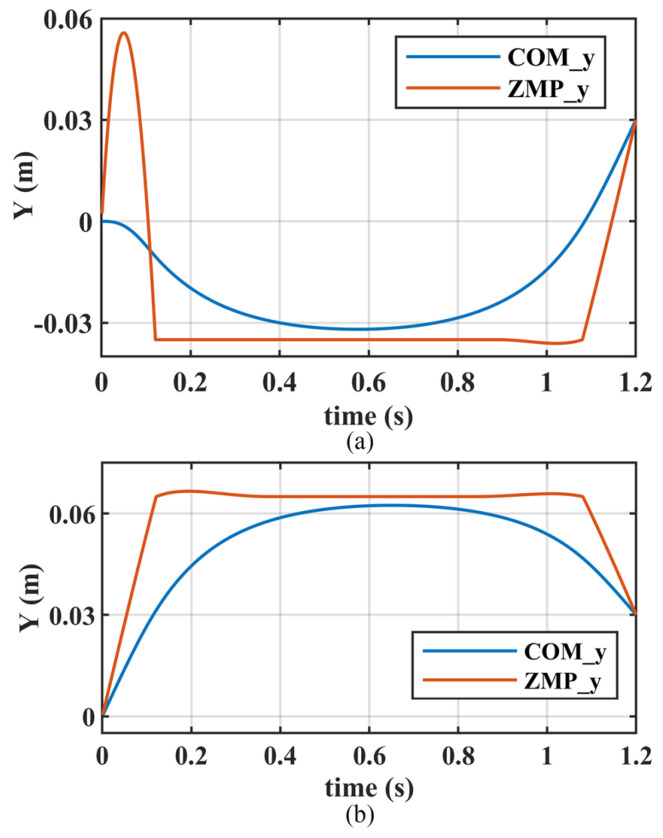
The COM and ZMP trajectories along the *Y*-axis for different support periods: (**a**) the first support period with an initial velocity of 0 m/s; (**b**) the second support period with an initial velocity of 0.28 m/s, which is the terminal velocity of the last support period.

Figure 13 illustrates the COM and ZMP trajectories along the *Y*-axis during the first and second support periods. The first support period is for right foot support and is the startup phase. In the IDS stage, ZMP first moves left to allow the COM to gain speed to the right, then moves right to enter the right foot support surface to achieve the transition to SS. The second support period is for left foot support. At the beginning of the period, the COM has a velocity to the left, which makes the trajectory of ZMP relatively smooth. Additionally, at the end of these two support periods, the COM has shifted 0.03 m to the left relative to its initial position. This illustrates that the step optimization proposed in this paper enables the robot to have lateral movement capability.

### 6.3. Walking Simulation on Stairs

**Figure 14:**
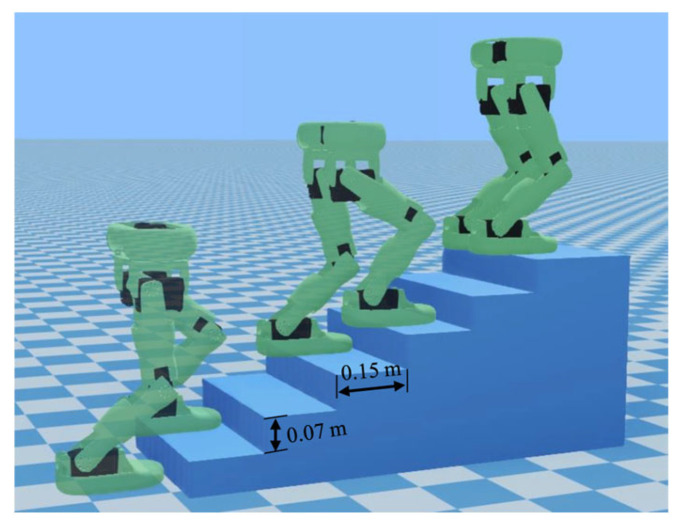
Simulation experiment for walking on stairs.

**Figure 15:**
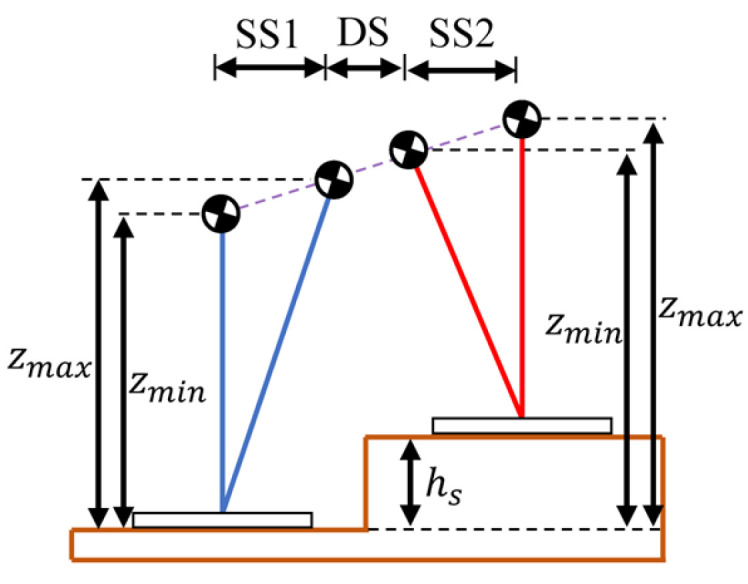
Schematic diagram of COM motion trajectory.

To validate the generation of walking patterns with height variation, the robot conducted a simulation experiment of climbing stairs, as shown in Figure 14. Figure 15 is a schematic diagram of COM motion trajectory with constraint. And the specified walking parameters are detailed in Table 5. Among them, the vertical range of COM motion is determined by the height of the step and the normal height of the COM.

**Figure 16:**
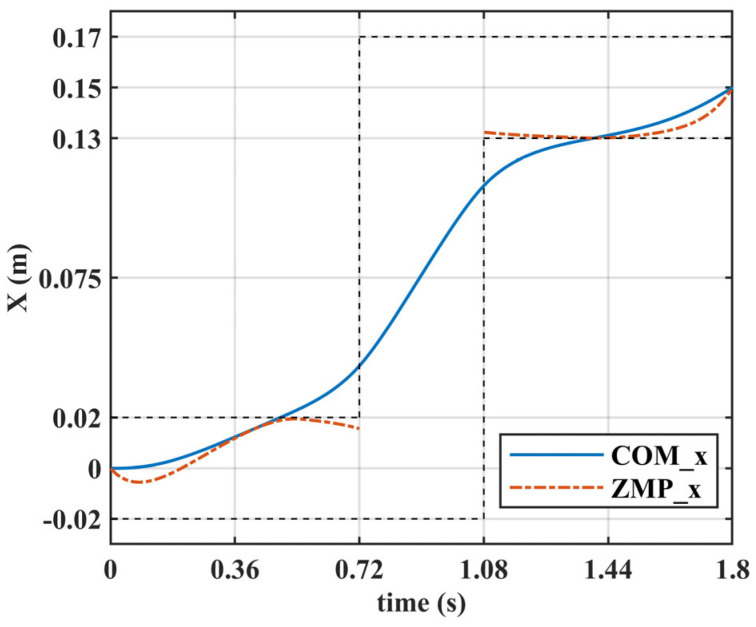
The COM and ZMP trajectories along the *X*-axis for the first step.

Figure 16 illustrates the COM and ZMP trajectories along the *X*-axis for the first step. During the single support phase, the ZMP is constrained within the support surface and remains near the edge at certain moments. This demonstrates the reliability of over-constraining the ZMP during changes in COM height.

**Figure 17:**
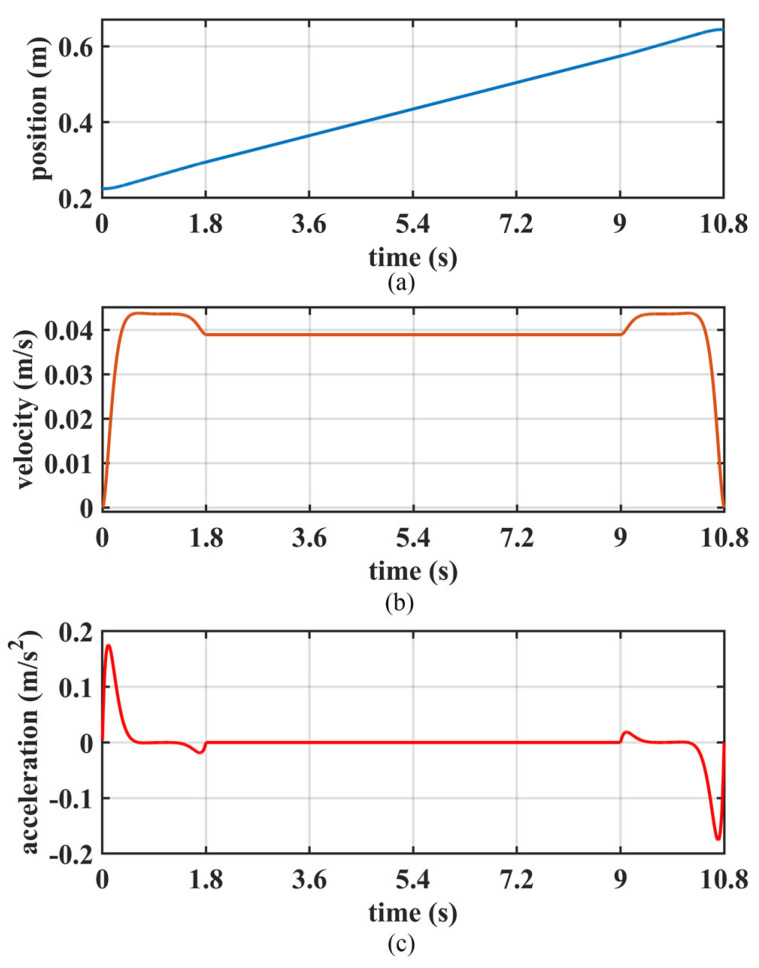
The COM motion trajectory along the Z-axis: (**a**) position, (**b**) velocity, and (**c**) acceleration.

The motion trajectory of COM on the *Z*-axis is shown in Figure 17. During the start step, the COM accelerates; during the stop step, it decelerates; and in the other steps, the COM moves upward at a constant speed.

**Figure 18:**
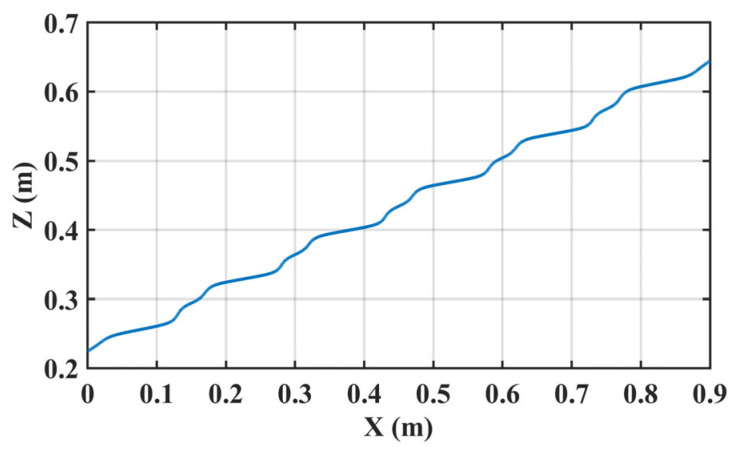
The motion trajectory of COM in the sagittal plane.

Figure 18 depicts the motion trajectory of the COM in the sagittal plane. In contrast to [26], this trajectory is not constrained to a straight line, making the movement of COM more flexible.

### 6.4. Walking Experiments

**Figure 19:**
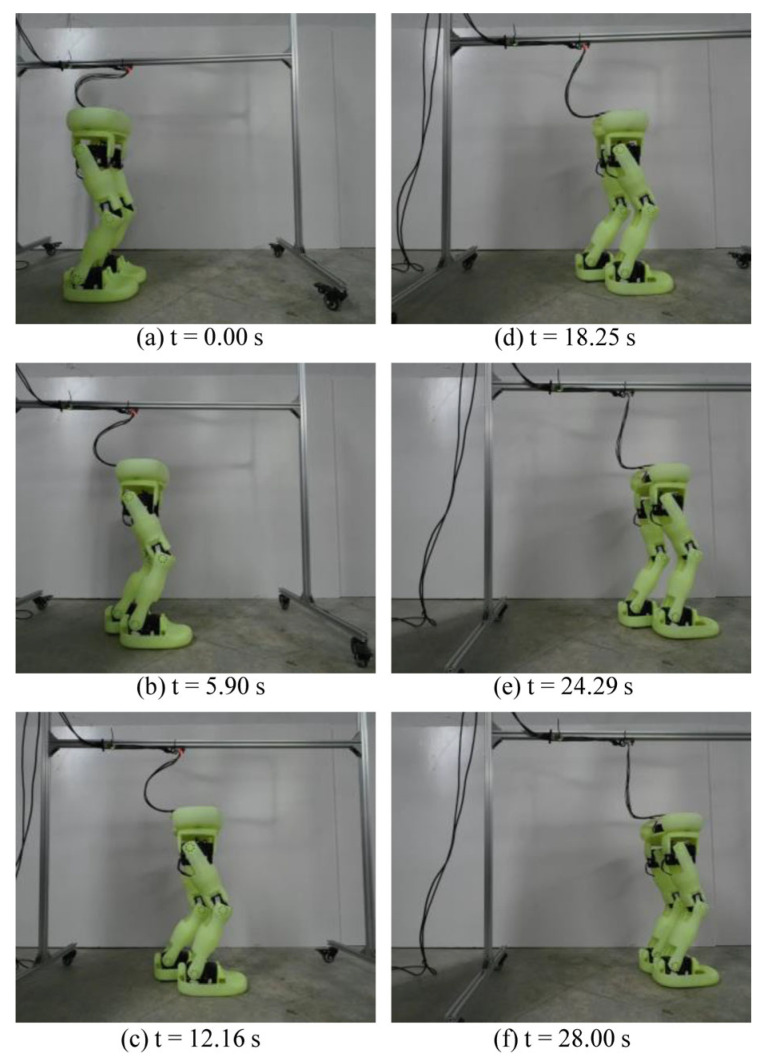
The experiment for walking in a straight line.

**Figure 20:**
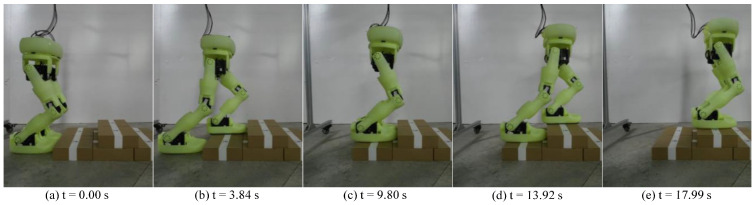
The experiment for walking on stairs.

The step optimization algorithm proposed in this article has also been verified on the real position-controlled biped robot Neubot. Figure 19 shows the walking experiment. Figure 20 shows the stair climbing experiment.

## 7. Conclusions

In this article, we focus on the generation of walking patterns for biped robots, and propose a novel method to generate the trajectory of the COM based on LIPM using quadratic optimization. First, this method employs a novel step that includes both the single support phase and the double support phase, enabling smooth transitions of the ZMP between the support feet. Second, by applying terminal constraints to the optimization problem, trajectories can be generated in units of a step, thereby shortening the optimization horizon. Third, this method achieves variations in the COM height while maintaining the linearity of the optimization problem, through excessive constraints on the ZMP within the support polygon. Furthermore, the performance of the proposed method is validated by simulations and experiments on a position-controlled biped robot, Neubot. Initially, the robot walked in a straight line. In the start, stop, uniform speed, and variable speed steps, the robot was able to obtain ideal COM and ZMP trajectories. Next, the robot walked diagonally. The trajectories of the COM and ZMP were closely aligned, ensuring the stability of the robot’s movement. Finally, the robot climbed stairs, achieving changes in the height of the COM. In future work, we plan to implement online generation of robot walking patterns, aiming to improve the robot’s adaptability to uneven terrain and its robustness against external perturbations [27,28,29,30,31].

## Figures and Tables

**Table 1:** A gait cycle of a human limb.

Stance		60%
Initial double stance	10%	
Single limb support	40%	
Terminal double stance	10%	
Swing		40%

**Table 2:** Walking parameters.

Step Period	Step Length	COM Height	Support Range	Without Double Support		With Double Support		
0.8 s	0.3 m	0.8 s	0.1 m	0.4 s	0.4 s	0.32 s	0.16 s	0.32 s

**Table 3:** The parameters for walking in a straight line.

Step Period	Step Length	COM Height	Support Range	SS1	DS	SS2
1.2 s	0.15 and 0.18 m	0.27 m	0.1 m	0.48 s	0.24 s	0.48 s

**Table 4:** The parameters for diagonal walking.

Step Period	$v_{x}$	$v_{y}$	COM Height	Support Range	SS1	DS	SS2
1.2 s	0.125 m/s	0.025 m/s	0.27 m	0.1 × 0.05 m	0.48 s	0.24 s	0.48 s

**Table 5:** The parameters for walking on stairs.

Step period T		1.8 s
COM normal height $z_{n}$		0.27 m
Step height $h_{s}$		0.07 m
Support range		0.2 × 0.2 m
Acceleration limit ${\ddot{z}}_{lim}$		0.1 × 9.8 $m/s^{2}$
SS1	Duration	0.4 ×T
	$p_{z}$	0
	$z_{min}$	$z_{n}$
	$z_{max}$	$z_{n}+0.5\times h_{s}$
DS	Duration	0.2 ×T
SS2	Duration	0.4 ×T
	$p_{z}$	$h_{s}$
	$z_{min}$	$z_{n}+0.5\times h_{s}$
	$z_{max}$	$z_{n}+h_{s}$

## Data Availability

The original contributions presented in this study are included in the article/Supplementary Material. Further inquiries can be directed to the corresponding author(s).

## Supplementary Materials

The following supporting information can be downloaded at: https://www.mdpi.com/article/10.3390/biomimetics10100654/s1, Video S1: Walking Pattern Generation through Step-by-Step Quadratic Programming for Biped Robots.
